# Role of soy isoflavone in preventing aging changes in rat testis: Biochemical and histological studies

**DOI:** 10.1016/j.sjbs.2022.103423

**Published:** 2022-08-18

**Authors:** Turki M. Al-Shaikh

**Affiliations:** Department of Biology, College of Science and Arts at Khulis, University of Jeddah, Jeddah, Saudi Arabia

**Keywords:** Aging, Ki-67 testicular immunoexpression, Testicular functions, Oxidative stress, Rat, Soy isoflavone, ELISA, The enzyme-linked immunosorbent assay, MDA, malonaldehyde, GSH, glutathione, SOD, superoxide dismutase, T, testosterone, SI, Soybean isoflavones, ANOVA, Analysis of variance, LSD, least significant difference, P, P-value, DHT, dihyrotestosterone, CAT, catalase, GST, glutathione transferase, GPx, glutathione peroxidase

## Abstract

Testicular function and structure harmed by ageing. Goal of this research was to assess preventive actions of soy isoflavone oral administration for 8 weeks on testes of old male albino rats, and potential mechanisms of action. Adult control (*N* = 10) and elderly control (*N* = 10) rats were fed usual diet, while aged treatment group (*N* = 10) gave oral 100 mg/kg soy isoflavone daily for 8 weeks. ELISA kits were used to measure testosterone levels and oxidative stress indicators [malonaldehyde (MDA), glutathione (GSH), and superoxide dismutase (SOD)] in serum. Aging produced functional and structural testicular changes and decreased ki67 proliferative marker immunoexpression versus adult control rats due to enhancement of oxidative stress. Soy isoflavone exerted protective effect on testicular function and structure as assessed by increase serum levels of testosterone and preserved histological structure and immune-expression features. These protected effects due to isoflavone antioxidant properties proved by decrease in serum values of MDA, while GSH and SOD were elevated after treatment. These data demonstrated protective effects of isoflavone against age changes in rat testes, by reducing oxidative stress and increasing antioxidants and testicular ki67 proliferative marker immunoexpression.

## 1 Introduction

Aging is a natural process that generates irreversible alterations in all body organs caused by environmental and endogenous factors. One consequence of ageing on the male reproductive system is changes in testicular morphology (Gunes et al., 2016). Recent research has connected prolonged paternal age to congenital malformations such as Down syndrome, cancers such as leukemia, CNS neoplasms, and a variety of neuropsychiatric disorders in kids (Gunes et al., 2016). Atrophic tubules were seen beside tubules with normal spermatogenesis in both humans and animals, indicating that aging-related atrophy begins in seminiferous tubules (Hussein et al., 2020).

The impact of advanced father age on the health of progeny have been extensively researched and summarized in recent researches (Belloc et al., 2014, Herati et al., 2017), but effects of age on male gonads (testes) are still poorly understood, and information is frequently disputed. Hypothalamic-pituitary–testicular axis (HPTA), that controls testicular functions affected by ageing. Spermatogenesis, an exocrine function, and steroidogenesis, an endocrine function, are the two primaries but not entirely independent testicular functions. Age-related changes in these processes cause changes in hormone values during senescence in animals and humans, including reduced levels of circulating androgens (Santiago et al., 2019).

Because of metabolism and cell replication high rates in testicular tissue, oxidative stress could be particularly destructive; thus, tissue antioxidant capacity is critical (Jalilvand et al., 2021). Antioxidant level may dramatically influence oxidative damaging effects related to advancing age and increased life duration. Age-associated testosterone (T) decreases is linked to clinical manifestations as sexual dysfunction, mood alterations, decreased muscle bulk, and an increased risk of cardiovascular disease, according to clinical investigations. As a result, enhancing testicular function in elderly men could be crucial to their sexual well-being (Pye et al., 2014). Hormone therapy is currently the most widely accepted treatment; nevertheless, the clinical usefulness and adverse actions of testosterone supplementation treatment are still debated, and the significance of other therapy is obvious (Huhtaniemi 2014). Soybean isoflavones (SI) are estrogen-like isoflavones that can be found in a variety of legume species. Soy flavonoids, genistein, and legumin are all found in SI. Soybean isoflavones had a broad spectrum of pharmacological benefits, as immune enhancement, anti-aging, anti-tumor, and other properties (Kuryłowicz 2021).

This research aimed to investigate the efficacy of Soy Isoflavone administration for 8 weeks on preservation of testicular functions and structure in aging male rats as well as possible mechanisms of action.

## 2 Materials and methods

### 2.1 Soy isoflavones

Soy isoflavone was get from I- Herb https://sa.iherb.com/pr/Nature-s-Plus-Ulta-Isoflavone-100–60-Vegetarian-Tablets/17613. Each tablet was dissolved in 10 ml of distal water and given orally to rats by gavage.

### 2.2 Animals and treatment

Ten adult male Albino rats (3–5 months) and twenty old aged (20–24 months) albino male rats utilized in this experimental research. Rats were getting from animal house at King Fahd Medical Research Center, Jeddah, Saudi Arabia. Animals sorted randamizely in separate cages (*N* = 5) and observed for one week for acclimatization to laboratory environment in controlled temperature (22–24 °C) with equal light- dark cycle. Rats were enable for free getting ordinal rats food and tap water *ad labium*. After acclimatization, rats were weighted and randomized divided into three groups. The National Institutes of Health's Guide for Care and Use of Laboratory Animals was followed throughout this research. Before experimenting on animals, the King Fahd Medical research Center Committee for Animal Care granted ethical approval. All attempts were made to ensure that the animals suffered as little as possible**.**

### 2.3 Experimental design

Rats sorted into three groups (10 rats each). Adult control and aged control groups, rats fed on basal chow food for 8 weeks. Aged treated group, rats administered 100 mg/kg of soy isoflavone each day by oral gavage feeding (Ustundag et al., 2007). Animals’ weights were estimated at the beginning and at the end of the study. Blood took from *retro*-orbital veins into a simple tube at the conclusion of the experiment. For 15 min, blood was centrifuged at 600 g for 10 min. Sera acquired and kept at −20 °C until needed.

### 2.4 Biochemical parameters

Rat's ELISA kits were purchase form Medical BioSource for estimation of serum levels of malonaldehyde (MDA) (catalog# MBS738685), glutathione (GSH) (catalog# MBS265966), superoxide dismutase (SOD) (catalog# MBS036924) and testosterone (catalog# MBS282195) based upon manufacture protocol.

### 2.5 Histological examination

Rats were killed after inhaling light diethyl ether anesthesia and cervical dislocation at the end of the trial. The testes were extracted, washed, and dried after the abdomen was dissected. Both sides had their testes extracted shortly. After weighing each testis (absolute weight), the relative testicular weight (testis weight divided by the body weight X 100) was calculated. For good tissue processing, formalin (10%) used to fix testicular tissues that had been changed several times. The tissues were cleaned in xylene after being graded and ethanol-treated. At 60 °C, the treated tissues were melted into paraffin. A rotary microtome machine was used to cut the paraffin blocks containing tissues into slices of about 5 µm thickness. Tissues were sliced and stained with hematoxylin and eosin after being placed on glass slides (H&E). Under a light microscope, stained tissue sections were photographed and structural changes were examined.

### 2.6 Immune staining

Antigen retrieval and chilling at room temperature followed deparaffinization in xylene and rehydration in ethanol at decreasing quantities. Endogenous peroxidase was suppressed by 10-minute incubation with 3 percent hydrogen peroxide at room temperature. The testicular tissue sections blocked in 10% normal goat serum for thirty minutes before being treated with primary antibody at 4 °C overnight. The primary antibody in this investigation was a rabbit monoclonal rabbit monoclonal anti Ki67 antibody at a dilution of 1:50 (Cat # ab16667; Abcam, Cambridge, USA).

### 2.7 Data analysis.

IBM SPSS Statistics for Windows, version 23 utilized to analyze data, which was expressed as mean ± standard deviation (IBM SPSS, IBM Corp., Armonk, N.Y., USA). The Shapiro–Wilk test employed to determine whether the value distribution was normal. To calculate significance, one-way ANOVA test then least significant difference (LSD) was used, assuming that the groups had identical variance. *P*-value <0.05 defined as statistical significance.

## 3 Results

### 3.1 Biological results

The initial and final body weights were significantly elevated in aged control (*P* < 0.0001 for both) and Aged isoflavone treated groups (*P* = 0.001 for both) versus adult control. While testicular index was significantly lower versus adult control (*P* < 0.0001 for both) (Table 1).

**Table 1:** Comparison of body weights and testicular weights and index between different studied groups.

Parameters	Adult control	Aged control	Aged Isoflavone
Initial body weight (grams)	189.40 ± 11.80	407.80 ± 13.94***	405.90 ± 56.01***
Final body weight (grams)	287.60 ± 12.35	419.30 ± 26.18***	418.60 ± 40.19***
Testicular weight (grams)	2.59 ± 0.58	2.61 ± 0.17	2.60 ± 0.27
Testicular index (%)	0.89 ± 0.04	0.63 ± 0.05***	0.62 ± 0.40***

*** Significance versus control adult.

### 3.2 Biochemical results

In this study, serum levels of testosterone were declined in aged control versus adult control (*P* < 0.0001 for both). However it is still significantly lower compared to adult control Meanwhile, after administration of isoflavone, testosterone serum versus increased in aged isoflavone group compared to aged control (*P* < 0.0001) (Table 2).

**Table 2:** Comparison of serum testosterone levels between different studied groups.

Parameters	Adult control (*n* = 10)	Aged control (*n* = 10)	Aged Isoflavone (*n* = 10)
Testosterone (ng/ml)	435.00 ± 21.44	200.70 ± 13.54***	381.50 ± 42.22***, ###

*** Significance versus control adult.

### Significance versus control aged.

In this study, GSH and SOD serum values were declined in aged control compared with adult control groups (*P* < 0.0001 for all). Meanwhile, administration of isoflavone led to significantly in GSH and SOD levels in aged isoflavone versus aged control (P = 0.015 and P = 0.035). MDA serum levels elevated in aged control versus adult control groups (*P* < 0.0001 for all) but was declined in aged isoflavone compared with aged control (*P* < 0.0001) (Table 3).

**Table 3:** Comparison of oxidative stress markers between different studied groups.

Parameters	Adult control (*n* = 10)	Aged control (*n* = 10)	Aged Isoflavone (*n* = 10)
GSH (ng/ml)	14.30 ± 0.95	10.00 ± 0.82***	13.10 ± 1.29***^,^###
SOD (U/ml)	172.90 ± 2.60	120.00 ± 1.83***	166.70 ± 10.36***^,^###
MDA (nmol/ml)	0.42 ± 0.02	0.97 ± 0.01***	0.73 ± 0.18***^,^###

GSH: glutathione; SOD: superoxide dismutase; MDA: malonaldehyde.

*** Significance versus control adult;

### Significance versus control aged.

### 3.3 Histological results

**Fig. 1:**
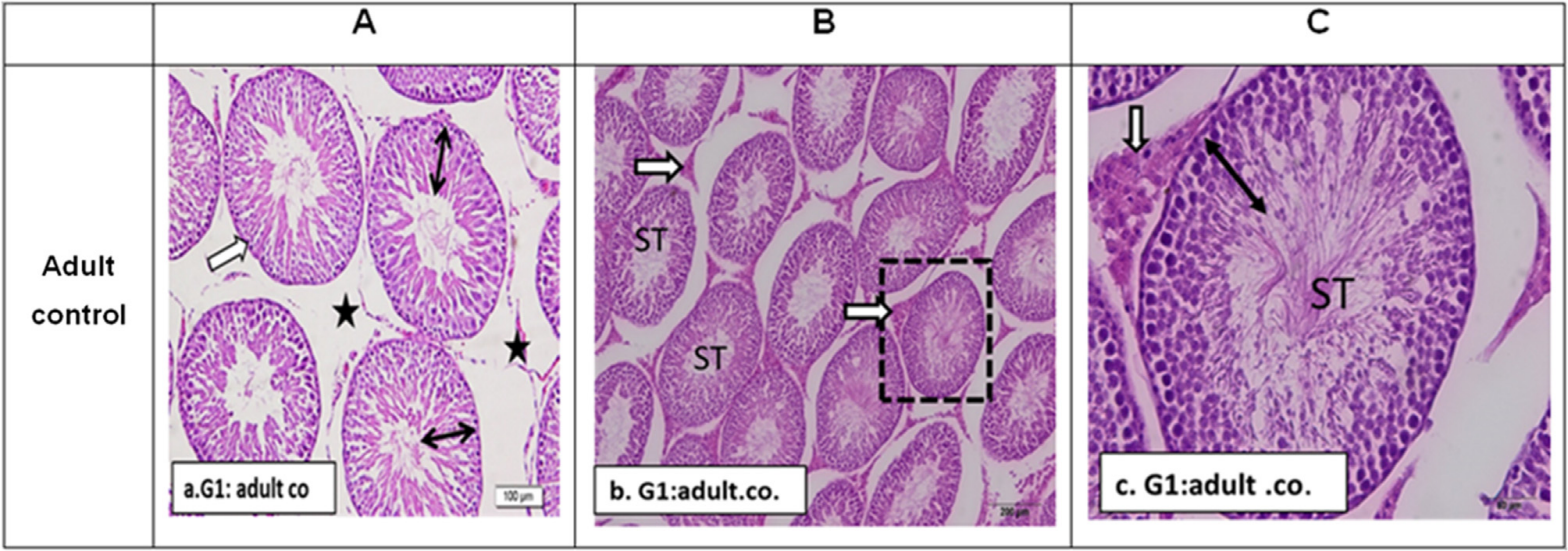
Sections in rat testis of G1: control rat stained by H&E to show: a. G1: adult control (3 months): showing normal seminiferous tubules with organized rounded outlines (white arrow), full thickness germ layer (double arrows). Most tubules contain mature sperms with their tails extending to the lumina of tubules. Interstitial cells and associated blood vessels were of normal density and appearance (stars). b. G1. Low power X100 to show seminiferous tubules (ST) with regular outlines and normal population of interstitial Leydig cells (white arrows). c. G1. Magnified power X400 of one seminiferous tubule (ST) to show full thickness germ cell layer (double head arrow) and normal Leydig cells with acidophilic cytoplasm and central nuclei (white arrow).

Fig. 1 shows typical seminiferous tubules with orderly rounded outlines and a full thickness germ layer in adult control rat testis. The testicular parenchyma was made up of numerous seminiferous tubules connected by thin interstitial gaps filled with loose areolar tissue. The majority of tubules had mature sperm with tails extending to tubule lumina. The density and appearance of interstitial cells and accompanying blood arteries were normal.

**Fig. 2:**
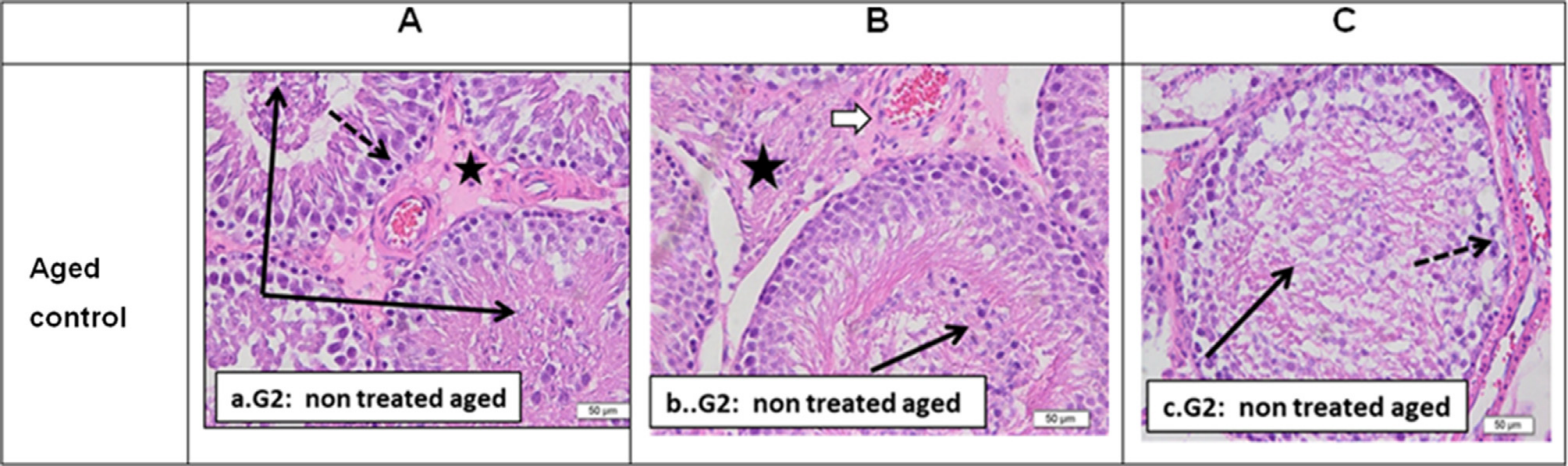
Sections from G2: Aged control non-treated rat testis (12 months) stained by H&E to show: a. G2. (X 400) powers showing seminiferous tubules with desquamation of degenerated cells into the lumen (black arrows). Interstitial tissue showed marked edematous deposition (homogenous acidophilic staining) congested blood vessels with dispersed Leydig cells (stars). Basal germinal cells showed small degenerated nuclei (dotted arrow). b. G2. Seminiferous tubules of another non treated aged rat (3 months) showing marked disruption and degenerative changes with few layers of degenerated germ cell. The lumina of tubules showed degenerated desquamated cells and residual sperms with clumped tails (black arrow). Interstitial blood vessel was thickened (white arrow) and surrounded by fibrous tissue with degenerated Leydig cells (star). c. G2. Another sample showing also marked complete loss of normal germ cell layers sparing the vasal layer which looked with dark small degenerated nuclei. Cell debris and clumped sperm tails could be seen within the lumen (black arrow). The basal lamina of the tubule looked thickened or surrounded by fibrosis (dotted arrow).

Aging results in alteration of normal seminiferous tubules structure of control non-treated rat testis (20–24 months) that revealed seminiferous tubules with desquamation of degenerated cells into the lumen and residual sperms with clumped tails. Interstitial tissue is widened and showed thick wall congested blood vessels with marked edematous deposition, and dispersed Leydig cells (Fig. 2).

**Fig. 3:**
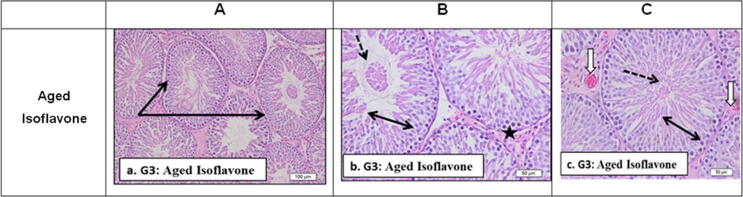
Sections from G3: aged rat (12 months) testis after soy isoflavone administration stained by H&E to show: a. G3: Showing marked preservation of normal testicular structure (Black arrows). b. G3. Showing seminiferous tubules with intact full thickness germ layers (double head arrow). Normal non edematous interstitial tissue and Leydig cells (black star). Few tubules showed desquamated cells (dotted arrows). c. G3. Similar preservation of normal seminiferous tubule outlines with full thickness germ layer (double head arrows). Mild interstitial edema with less thickened blood vessels (white arrows) compared to non-treated aged rats.

Administration of soy isoflavone markedly prevents progress in aging changes as shown in Fig. 3 that revealed marked preservation of normal testicular structure as seminiferous tubules appeared with intact full thickness germ layers, normal non-edematous interstitial tissue and Leydig cells. Only few tubules showed desquamated cells, mild interstitial edema with less thickened blood vessels compared to non-treated aged rats.

### 3.4 Immunohistochemistry stains results

**Fig. 4:**
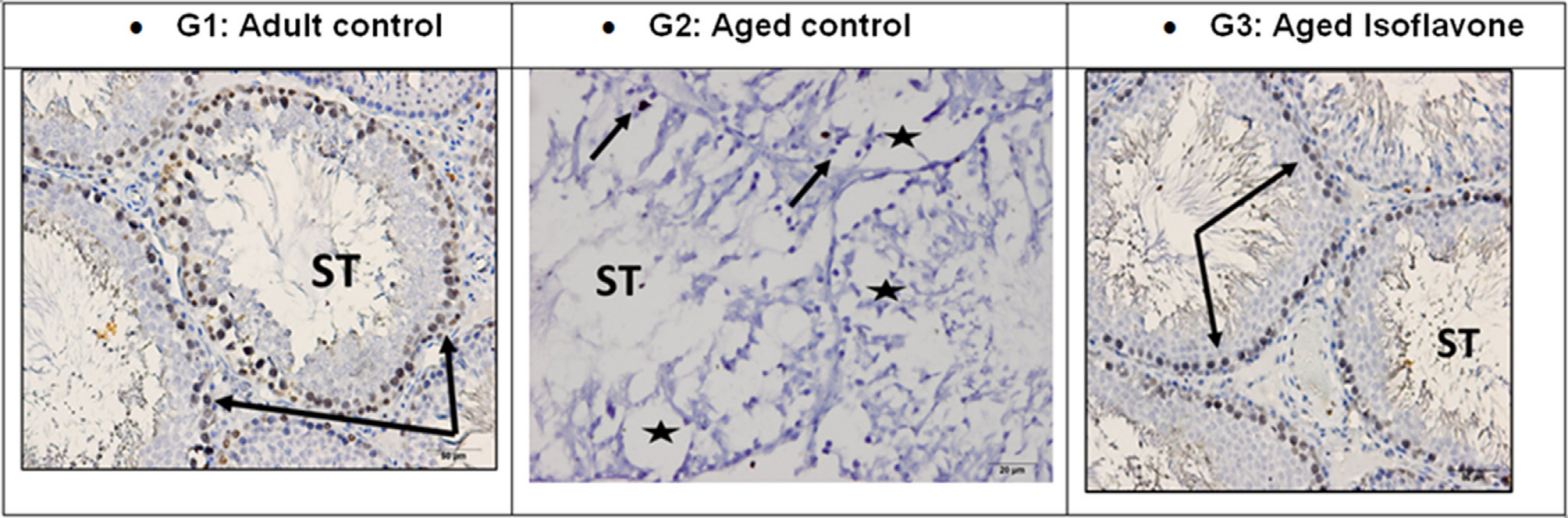
Sections from male rat testis immune-expression for KI67 marker in the seminiferous tubules (ST) cells showing: G1: Normal adult control testis (3 months) showing high immune- positive reaction for Ki67 in the seminiferous tubules basal spermatogenic layers (spermatogonia and primary spermatocytes and occasionally secondary spermatocytes (black arrows). G2: Aged control aged testis (12–18 months) showing seminiferous tubules with disorganized degenerated germ layers and presence of many spaces (stars) indicated degenerated cells. Ki 67 immuno-expression is nearly absent except of one or two degenerated nuclei looked positive (black arrows). G3: Aged testis after 30 days of isoflavone administration showing immuno-positive reaction in the basal spermatogonia layer (black arrows).

Immunostaining of testicular tissue for Ki-67; the marker for cell proliferation showed marked decrease or loss of positive immunostaining in the degenerated seminiferous tubules of G2 aged rats compared to G1: control adult which showed in the high immune -expression in the basal layers of spermatogenic cells (spermatogonia., primary spermatocytes and occasionally secondary spermatocytes). In G3: aged treated with isoflavone extract restoration of ki-67 expression especially basal layers could be observed Fig. 4.

## 4 Discussion

Aging is an irreversible process that occurs naturally in biological organisms as they mature. According to studies, ageing inhibits male reproductive function and causes senile fathers to produce defective sperm, resulting in offspring with elevated prevalence of genetic defects, pediatric cancers, and a variety of neuropsychiatric illnesses (Gunes et al., 2016). The results of this research revealed that aging led to decreased in serum testosterone level compared with adult male rats. Meanwhile, testosterone serum levels significantly increased in aged isoflavone treated rats versus aged rats without treatment, but the level still low than adult male rats. In this respect, (Yi et al., 2002) reported elevation in serum androgen values and decline in dihydrotestosterone (DHT) in isoflavone treated rats versus isoflavone free control. (Fritz et al., 2002) reported that rats (aged 70 days) treated with genistein (25 and 250 mg/kg) for their whole life showed higher blood testosterone values versus genistein-free controls. (Dalu et al., 2002) reported that old male rats (140 days) fed on isoflavones (500 mg/ kg) had raised serum androgens. (Li et al., 2017) found that obese male rats given soya isoflavone (SIF) at doses of 0, 50, 250, and 500 mg/kg/day for 4 weeks had reduced body weight in a dose-dependent way, reduced testicular damage, and increased testosterone levels, as well hydroxysteroid dehydrogenase-3β (HSD3β), steroidogenic acute regulatory (StAR), cytochrome P450 17A1 (CYP17), cytochrome P450 11A1 (CYP11A1), hydroxysteroid dehydrogenase-17β (HSD17β) protein and mRNA levels.

Organ damage is largely caused by oxidative stress. By catalyzing dismutation of superoxide anion to H_2_O_2_, that then processed by catalase or glutathione peroxidase, SOD acts as first gatekeeper in antioxidant defense system as glutathione peroxidase (GPx). Glutathione (GSH), an important redox buffer, is co-substrate for glutathione transferase (GST) in biochemical xenobiotic conjugation, and MDA is important result of membrane lipid peroxidation and is an ageing marker (Oyebode et al., 2020). In this work, an imbalance between antioxidative and oxidative markers exposed ageing testis to oxidative stress, resulting in testicular regression in this group. Apoptosis of germ cells in old age is linked to an oxidative/antioxidative imbalance (Beattie et al., 2013). With age, the enzymatic defense systems that protect the germinal epithelium can no longer deal with significant oxidative destruction, and apoptotic changes, particularly in germ cells, rise. As a result, ageing testes experience significant histological and morphological changes, resulting in decreased testicular function (Matzkin et al., 2016). According to (Hussein et al., 2020) the most essential markers for sustaining the cells' redox status are assessed NO, MDA, and GSH in testicular tissue. Senescence in reproductive cells causes the formation of oxygen free radicals in Leydig cells and sperm mitochondria, led to disruption of sperm telomere, Leydig cells steroidogenesis, and presence of mitochondrial DNA in both cells (Desai et al., 2010).

The testis of the aged non-treated rat group revealed modest histological alterations in seminiferous tubules, including desquamation of degenerated cells into lumen and remnant sperms with clumped tails, in this study. Edematous interstitial tissue with scattered Leydig cells and crowded thick-walled arterioles with narrowing lumens. There was also a noticeable expansion of interstitial spaces, which were closed by Leydig cells' cytoplasmic vacuolization. The current study's histopathologic changes corroborate prior research that found comparable age-related changes (Vickers, 2017). Germ cell loss was linked to the existence of seminiferous epithelial vacuoles (Pop et al., 2011), while abnormal germ cells phagocytosed by Sertoli cells linked to Sertoli cell cytoplasm vacuolization (Gunes et al., 2016). With ageing, the presence of vacuoles suggested a decline in Sertoli cell biological activity (Jiang et al., 2014, Santiago et al., 2019). Ultrastructural findings revealed expanded areas next to basement membranes, indicating spermatogonia germ cell loss, as well as substantial vacuolization of the neighboring Sertoli cells, indicating that seminiferous epithelium vacuolization is multifactorial. The occurrence of phagocytosed necrotic germ cells in Sertoli cell cytoplasm raised the possibility that this was one of reasons of Sertoli cell vacuolization. In elderly adults, disruption of the Sertoli-to-Sertoli junction associated to abnormal spermatogenesis and weakening of immunological barrier provided by blood testis barrier (Jiang et al., 2014, Santiago et al., 2019). In concomitant with histopathological alterations observed in (Hussein et al., 2020) research, in senile Brown Norway rats, regressed tubules with degraded germ cells and wide intercellular gaps were discovered (LEVY et al., 1999). One interesting result noted in (Hussein et al., 2020) study was elevated in Leydig cells number in interstitium around atrophic tubules. Other investigators found elevated in Leydig cells number and size with aging (Dakouane et al., 2005, Xu et al., 2013). The elevated size and number of Leydig cells revealed a compensatory action of aged testicular tissue, unfortunately nonfunctioning.

Ki-67 immunohistochemistry was used in this study to confirm and investigate germ cell proliferative activity in aged seminiferous tubules. This marker was found to be significantly lower in aged samples compared to adult rats. KI-67 identified and utilized as a cell proliferation indicator (Scholzen and Gerdes 2000). Bullwinkel et al. (2006) reported that ribosomal RNA transcription needed for cellular activity and protein synthesis for cellular division linked to Ki-67 protein. Its decrease in the present study in ageing seminiferous tubules go in hand with what was mentioned by Rahmanzadeh et al. (2007), that antigen KI-67 inactivation inhibits ribosomal RNA formation and hence stoppage of germ cell regeneration seen in the present study. Soy bean isoflavone seemed to work on Ki-67 preventing its down regulation by aging processes and thus maintaining viability of seminiferous tubules germ cells especially early stages mainly spermatogonia and primary spermatocytes.

## 5 Conclusions

The current study's findings revealed the occurrence of age-related regressive alterations in testes, as evidenced by histological and biochemical results. The biochemical data and immunohistochemistry expression of Ki-67 in aged testis revealed disturbance between oxidative damage and anti-oxidant defense, as well as alterations in germ cells and Leydig cells that were reversed by isoflavone treatment for 8 weeks.

## Declaration of Competing Interest

The authors declare that they have no known competing financial interests or personal relationships that could have appeared to influence the work reported in this paper.

## References

[b0005] Beattie M.C., Chen H., Fan J., Papadopoulos V., Miller P., Zirkin B.R. (2013). Aging and luteinizing hormone effects on reactive oxygen species production and DNA damage in rat leydig cells. Biol. Reprod..

[b0010] Belloc S., Hazout A., Zini A., Merviel P., Cabry R., Chahine H., Copin H., Benkhalifa M. (2014). How to overcome male infertility after 40: Influence of paternal age on fertility. Maturitas.

[b0015] Bullwinkel J., Baron-Lühr B., Lüdemann A., Wohlenberg C., Gerdes J., Scholzen T. (2006). Ki-67 protein is associated with ribosomal rna transcription in quiescent and proliferating cells. J. Cell. Physiol..

[b0020] Dakouane M., Bicchieray L., Bergere M., Albert M., Vialard F., Selva J. (2005). A histomorphometric and cytogenetic study of testis from men 29–102 years old. Fertil. Steril..

[b0025] Dalu A., Blaydes B.S., Bryant C.W., Latendresse J.R., Weis C.C., Delclos K.B. (2002). Estrogen receptor expression in the prostate of rats treated with dietary genistein. J. Chromatogr. B.

[b0030] Desai N., Sabanegh E., Kim T., Agarwal A. (2010). Free radical theory of aging: Implications in male infertility. Urology.

[b0035] Fritz W.A., Wang J., Eltoum I.-E., Lamartiniere C.A. (2002). Dietary genistein down-regulates androgen and estrogen receptor expression in the rat prostate. Mol. Cell. Endocrinol..

[b0040] Gunes S., Hekim G.N.T., Arslan M.A., Asci R. (2016). Effects of aging on the male reproductive system. J. Assist. Reprod. Genet..

[b0045] Herati A.S., Zhelyazkova B.H., Butler P.R., Lamb D.J. (2017). Age-related alterations in the genetics and genomics of the male germ line. Fertil. Steril..

[b0050] Huhtaniemi I. (2014). Late-onset hypogonadism: Current concepts and controversies of pathogenesis, diagnosis and treatment. Asian J. Androl..

[b0055] Hussein S.M., El-Fadaly A.B., Metawea A., Khaled B. (2020). Aging changes of the testis in albino rat: Light, electron microscopic, morphometric, immunohistochemical and biochemical study. Folia Morphol..

[b0060] Jalilvand N., Hosseini M., Beheshti F., Ebrahimzadeh-Bideskan A. (2021). Protective effect of pparγ agonist pioglitazone, on testicular tissue and sperm parameters in hypothyroid rats. Toxin Rev..

[b0065] Jiang H., Zhu W.-J., Li J., Chen Q.-J., Liang W.-B., Gu Y.-Q. (2014). Quantitative histological analysis and ultrastructure of the aging human testis. Int. Urol. Nephrol..

[b0070] Kuryłowicz A. (2021). The role of isoflavones in type 2 diabetes prevention and treatment—a narrative review. Int. J. Mol. Sci..

[b0075] Levy S., Serre V., Hermo L., Robaire B. (1999). The effects of aging on the seminiferous epithelium and the blood—testis barrier of the brown norway rat. J. Androl..

[b0080] Li L., Chen X., Luo Q., Huang C., Liu W., Chen Z. (2017). Effects of soy isoflavones on testosterone synthetase in diet-induced obesity male rats. Int. J. Clin. Exp. Path..

[b0085] Matzkin M.E., Miquet J.G., Fang Y., Hill C.M., Turyn D., Calandra R.S., Bartke A., Frungieri M.B. (2016). Alterations in oxidative, inflammatory and apoptotic events in short-lived and long-lived mice testes. Aging (Albany NY).

[b0090] Oyebode O.T., Giwa O.D., Olorunsogo O.O. (2020). Comparative effects of galactose-induced aging on mitochondrial permeability transition in rat liver and testis. Toxicol. Mech. Methods.

[b0095] Pop O., Cotoi C.G., Plesea I., Enache S., Popescu F.C., Enache M., Plesea R. (2011). Correlations between intralobular interstitial morphological changes and epithelial changes in ageing testis. Rom. J. Morphol. Embryol..

[b0100] Pye S.R., Huhtaniemi I.T., Finn J.D., Lee D.M., O'Neill T.W., Tajar A., Bartfai G., Boonen S., Casanueva F.F., Forti G., Giwercman A., Han T.S., Kula K., Lean M.E., Pendleton N., Punab M., Rutter M.K., Vanderschueren D., Wu F.C.W. (2014). Late-onset hypogonadism and mortality in aging men. J. Clin. Endocrinol. Metabolism.

[b0105] Rahmanzadeh R., Hüttmann G., Gerdes J., Scholzen T. (2007). Chromophore-assisted light inactivation of pki-67 leads to inhibition of ribosomal rna synthesis. Cell Prolif..

[b0110] Santiago J., Silva J.V., Alves M.G., Oliveira P.F., Fardilha M. (2019). Testicular aging: An overview of ultrastructural, cellular, and molecular alterations. J. Gerontol.: Ser. A.

[b0115] Scholzen T., Gerdes J. (2000). The ki-67 protein: From the known and the unknown. J. Cell. Physiol..

[b0120] Ustundag B., Bahcecioglu I.H., Sahin K., Duzgun S., Koca S., Gulcu F., Ozercan I.H. (2007). Protective effect of soy isoflavones and activity levels of plasma paraoxonase and arylesterase in the experimental nonalcoholic steatohepatitis model. Dig. Dis. Sci..

[b0125] Vickers N.J. (2017). Animal communication: When i’m calling you, will you answer too?. Curr. Biol..

[b0130] Xu Y.-C., Jing L., Liang W.-B., Zhu W.-J. (2013). Evaluation on changes of testicular histology in aging men. J. Reprod. Contracept..

[b0135] Yi M.-A., Son H.M., Lee J.-S., Kwon C.-S., Lim J.K., Yeo Y.K., Park Y.S., Kim J.-S. (2002). Regulation of male sex hormone levels by soy isoflavones in rats. Nutr. Cancer.

